# Control of fear extinction by hypothalamic melanin-concentrating hormone–expressing neurons

**DOI:** 10.1073/pnas.2007993117

**Published:** 2020-08-26

**Authors:** Cristina Concetti, Edward F. Bracey, Daria Peleg-Raibstein, Denis Burdakov

**Affiliations:** ^a^Department of Health Sciences and Technology, ETH Zürich, 8603 Schwerzenbach, Switzerland;; ^b^Institute for Neuroscience, Department of Health Sciences and Technology, ETH Zürich, 8603 Schwerzenbach, Switzerland;; ^c^Institute of Food Nutrition and Health, Department of Health Sciences and Technology, ETH Zürich, 8603 Schwerzenbach, Switzerland;; ^d^Neuroscience Center Zürich, 8057 Zürich, Switzerland

**Keywords:** fear, hypothalamus, learning, MCH

## Abstract

Learning to fear danger is essential for survival. However, overactive, relapsing fear behavior in the absence of danger is a hallmark of disabling anxiety disorders that affect millions of people. Its suppression is thus of great interest, but the necessary brain components remain incompletely identified. We studied fear suppression through a procedure in which, after acquiring fear of aversive events (fear learning), subjects were exposed to fear-eliciting cues without aversive events (safety learning), leading to suppression of fear behavior (fear extinction). Here we show that inappropriate, learning-resistant fear behavior results from disruption of brain components not previously implicated in this disorder: hypothalamic melanin-concentrating hormone–expressing neurons (MNs). Using real-time recordings of MNs across fear learning and extinction, we provide evidence that fear-inducing aversive events elevate MN activity. We find that optogenetic disruption of this MN activity profoundly impairs safety learning, abnormally slowing down fear extinction and exacerbating fear relapse. Importantly, we demonstrate that the MN disruption impairs neither fear learning nor related sensory responses, indicating that MNs differentially control safety and fear learning. Thus, we identify a neural substrate for inhibition of excessive fear behavior.

## Significance

Excessive fear is a hallmark of disabling anxiety disorders that affect millions of people worldwide. Its suppression is thus of great interest, but the necessary brain components remain incompletely identified. We found that inappropriate, learning-resistant fear results from disruption of brain components not previously implicated in this disorder: hypothalamic melanin-concentrating hormone–expressing neurons (MNs). We show that the brain converts fear-inducing events into MN signals. These brief signals are necessary for healthy long-term flexibility of fear behavior. Without them, a fearful experience leads to abnormal treatment-resistant, relapsing fear reminiscent of posttraumatic stress disorder. These findings identify natural brain signals that suppress excessive fear behavior.

Developing fear responses to dangerous situations is a vital evolutionary adaptation across the animal kingdom, which confers survival advantages by helping to avoid danger. However, when danger is no longer likely, continued fear responses are maladaptive since they can disable an individual’s ability to function in society without providing any advantage. Indeed, persistent fear response in the absence of danger is a hallmark of many disabling anxiety disorders that affect millions of people worldwide (1–3).

Posttraumatic stress disorder (PTSD), an anxiety disorder, is characterized by a resistance to safety learning (4). Approximately 8% of the general population in the United States develop PTSD at some time in their lives (5), and 90% are exposed to one or more traumatic events, but most do not go on to develop PTSD (6). Furthermore, from a clinical perspective, the return of fear after extinction is thought to contribute to relapse following exposure-based therapies for anxiety disorders (7). PTSD can be treated by psychotherapeutic interventions and by pharmacologic treatments (8, 9); however, the long-term efficacy of these treatments is challenged by the propensity of extinguished fear to relapse (1, 10, 11). Therefore, gaining a better understanding of brain mechanisms that suppress overactive, relapsing fear is of continuing high interest.

Pavlovian fear conditioning is one of the leading translational models for studying fear acquisition and subsequent suppression by extinction-based exposure therapy (12). In this procedure, after acquiring fear of aversive events (fear learning), subjects are exposed to fear-eliciting cues without aversive events (safety learning), leading to suppression of fear behavior (fear extinction) (1, 12). How the brain prevents overactive fear behavior without compromising the useful fear learning is poorly understood. Safety learning is thought to update cue→behavior coupling without erasing fear learning and memory (1, 13, 14); however, the brain components that regulate safety learning without interfering with fear learning remain incompletely identified.

Research on fear conditioning and extinction has traditionally focused on such brain areas as the amygdala, prefrontal cortex, and hippocampus (15, 16). In contrast, the role of the hypothalamus has remained relatively underexplored, despite decades of studies implicating this brain area in memory disorders (17–20). In particular, neurons expressing the peptide neurotransmitter melanin-concentrating hormone (MNs), which are found exclusively in the lateral hypothalamus, innervate many brain areas implicated in safety learning (20, 21). Recent experimental evidence has led to the suggestion that MN activity may promote multiple forms of synaptic and behavioral flexibility through either learning or forgetting (20, 22–25). However, it is unknown if and when the endogenous MN activity regulates fear learning and/or extinction. The present study aimed to define endogenous MN activation patterns across the multiple phases of fear learning and extinction, and to test whether MN activity is causally linked to specific features of overactive fear behavior.

## Results

### MN Activity during Acquisition and Extinction of Fear.

**Fig. 1.**
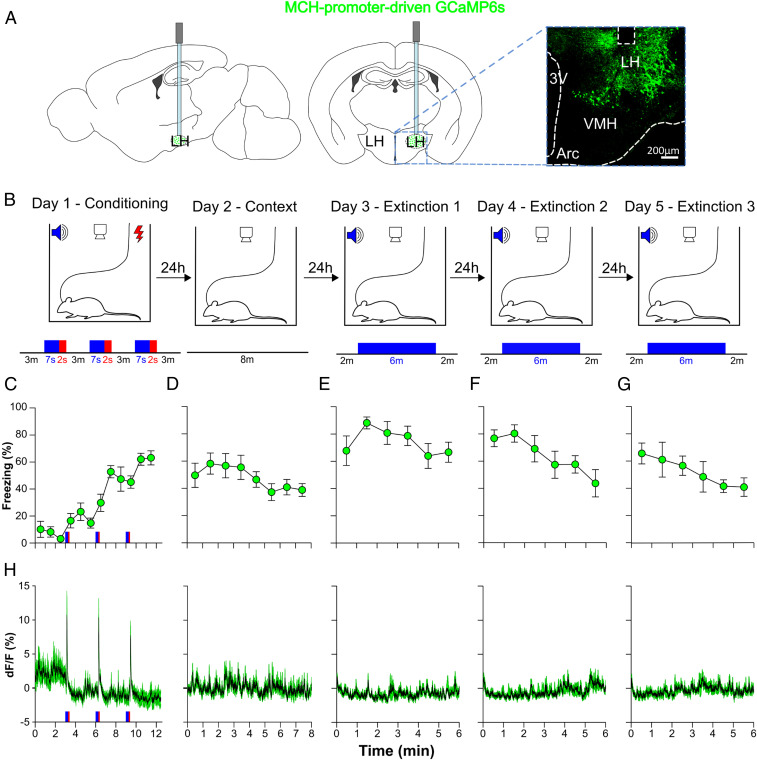
MN activation pattern during fear conditioning and extinction. (*A*) Targeting schematic (*Left*) and expression (*Right*) of GCaMP6s in MNs. The dashed square box indicates fiber location. (*B*) Protocol of fear conditioning and extinction. Tone, blue; shock, red. (*C*) Freezing during conditioning. Each point shows a 60-s average (30 s before and 30 s after the point). (*D*) Freezing to context alone. (*E*–*G*) Freezing during tones in fear extinction. (*H*) MN-GCaMP6s activity recorded concurrently (in the same mice) with behavior shown in *C*–*G*. Data are mean ± SEM of seven MN-GCaMP6s mice.

We performed fear conditioning and extinction while recording hypothalamic MN activity using fiber photometry of the genetically targeted calcium indicator GCaMP6s (Fig. 1*A* and *B*). On day 1, mice were exposed to three 7-s tones with a 2-s foot shock at the end of the tones (conditioning). On day 2, mice were placed back in the conditioning cage for 8 min to quantify fear induced by the cage without any cues (context test). On days 3 to 5, mice were exposed to the same tone for 6 min in the absence of foot shock (extinction). As a behavioral readout of fear, we quantified freezing (26, 27). During conditioning (Fig. 1*C*), mice froze little before and during the first tone, but showed increased cued freezing (freezing in response to tone) across the repeated tone-shock pairings, indicating fear learning (*SI Appendix*, Fig. S1*A*). Mice displayed moderate freezing during the context test (Fig. 1*D*). During the extinction days (Fig. 1 *E*–*G*), mean freezing decayed significantly between sessions, indicating fear extinction (*SI Appendix*, Fig. S1*B*). These data confirm that mice acquired and extinguished cued fear as expected (26, 27).

**Fig. 2.**
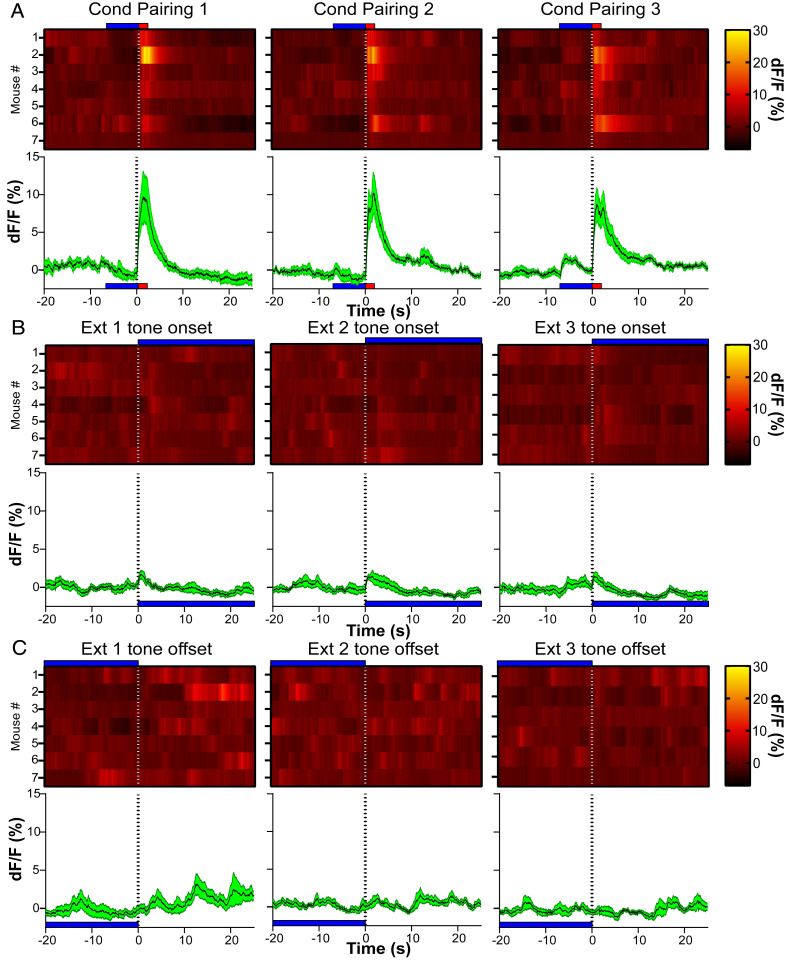
Effects of shocks and tones on MN activity. (*A*) Shock and tone-associated MN-GCaMP6s activity during conditioning. (*B*) Tone onset-associated MN-GCaMP6s activity during extinction. (*C*) Tone offset-associated MN-GCaMP6s activity during extinction. In *A*–*C*: (*Top*) Heatmaps of normalized MN-GCaMP6s fluorescence of individual mice, aligned to shocks; (*Bottom*) corresponding group data (mean ± SEM).

We then analyzed behavior and cue-aligned MN-GCaMP6s signals to determine whether and when MN activation occurs during fear conditioning and extinction. In MNs, GCaMP6s fluorescence is a good proxy for activity, because it is linearly related to MN action potential firing frequency (28). During fear conditioning, we observed a rapid and pronounced rise of GCaMP6s fluorescence during foot shocks (Figs. 1*H* and 2*A*); the shock-induced MN-GCaMP6s signal was 9.10 ± 2.65% (one-sample *t* test, *P* = 0.0139, *n* = 7 mice). The signal rise was associated with shock onset rather than tone offset (*SI Appendix*, Fig. S2*A*). In contrast, no MN-GCaMP6s activation in response to tones was found during either conditioning or extinction. For conditioning, the mean peak tone-evoked MN-GCaMP6s signal was 0.00 ± 0.39% (one-sample *t* test, *P* = 0.8749; *n* = 7 mice) (Fig. 2*A*). For extinction, the peak tone onset-evoked MN-GCaMP6s signal was 0.04 ± 0.03% (one-sample *t* test, *P* = 0.2013) and tone offset was 0.03 ± 0.05% (one-sample *t* test, *P* = 0.5543; *n* = 7 mice) (Fig. 2 *B* and *C*). These results suggest that across fear conditioning and extinction, endogenous bursts of MN activity are evoked primarily by conditioning shocks.

To control for possible artifacts in these recordings, we additionally analyzed the raw calcium-independent MN-GCaMP6s fluorescence signal evoked by isosbestic 405-nm excitation; this control signal reports such events as movement artifacts (29). At the time of shock, there was a slight depression in this signal, which was the opposite of the increased fluorescence observed at 465-nm excitation (*SI Appendix*, Fig. S2*B* and *Methods*). This suggests that our observations are not due to recording artifacts but rather reflect endogenous MN activity. In theory, such endogenous MN bursts may affect concurrent behavior and/or future cue→behavior coupling (25). To discriminate between these possibilities, we next sought to establish causal roles of shock-associated endogenous MN activity in the multiple phases of fear behavior.

### Role of MNs in Fear Extinction.

**Fig. 3.**
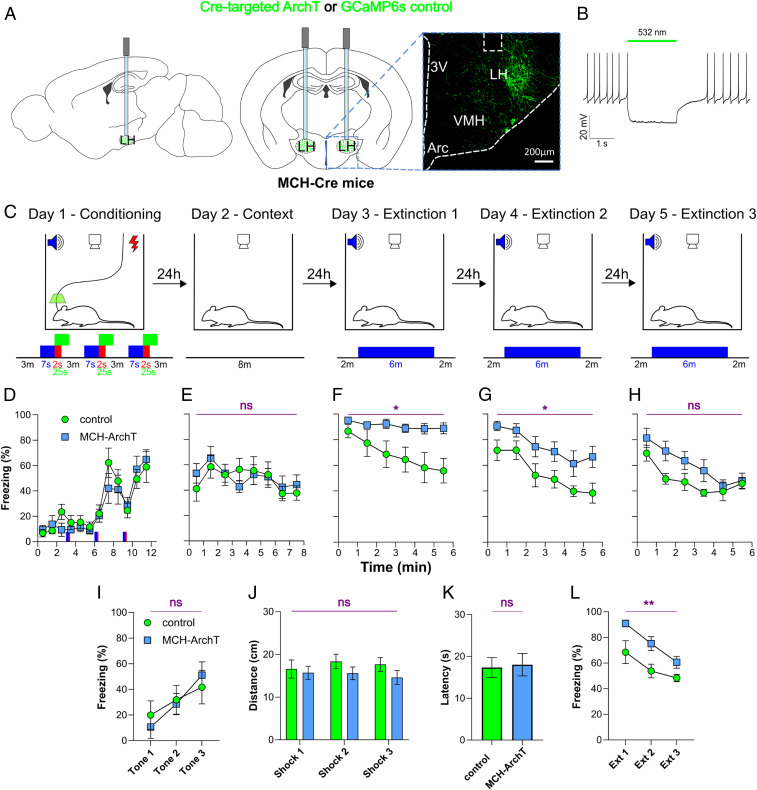
Effects of MN photoinhibition on fear behavior during conditioning and extinction. (*A*) Targeting schematic (*Left* and *Center*), and typical expression of ArchT (*Right*) in MNs. The dashed white box indicates fiber location. (*B*) Patch-clamp recording from acute hypothalamic brain slices confirming silencing of MN-ArchT cells by green light (representative response of *n* = 10 cells). (*C*) Behavioral protocol showing temporal targeting of MN photoinhibition. Blue, tone; red, shock; green, laser activation. (*D*–*H*) Effect of MN photoinhibition on freezing corresponding to test phases shown in *C*. (*I*) Effect of MN photoinhibition on mean freezing during 7-s tones on each conditioning day. (*J*) Effect of MN photoinhibition on distance moved during shocks. (*K*) Effect of MN photoinhibition (during hot plate test) on paw withdrawal latency. (*L*) Effect of MN photoinhibition on mean freezing during each 6-min extinction session. Data are mean ± SEM of seven MN-ArchT mice and six control mice. ns, *P* > 0.05; *P < 0.05; **P < 0.01 for ArchT vs. control. More details on the statistical tests are provided in *Results*.

To explore whether the shock-associated MN activation influences fear behavior, we used optogenetics to inhibit MNs with temporal specificity. We targeted an optogenetic inhibitory actuator (ArchT; *n* = 7 mice) or a control virus (GCaMP6s; *n* = 6 mice) to MNs and bilaterally implanted optic fibers in the lateral hypothalamus (Fig. 3*A* and *Methods*). We confirmed effective MN-ArchT photoinhibition using patch-clamp recordings in acute brain slices (Fig. 3*B*). Control experiments, based on reports that chronic MN deficiency produces weight loss and hyperlocomotion (30, 31), showed that body weight and baseline locomotion of MN-ArchT mice were indistinguishable from that of control mice (*SI Appendix*, Fig. S3 *A*–*C*). The baseline (i.e., in the absence of green laser illumination) biophysical properties of MN-ArchT neurons and MN numbers were also similar to those in control brains (*SI Appendix*, Fig. S3 *E* and *F*). This confirms that ArchT transgene does not disrupt MN physiology and MN-dependent organismal parameters in the absence of photoinhibition.

To inhibit MNs during the time when they displayed shock-associated activation, lateral hypothalamic photoillumination (bilateral 532-nm laser) was applied for 25 s at the onset of each conditioning shock (Fig. 3*C*). During the fear conditioning session (Fig. 3*D*), MN-ArchT mice with the photoinhibited shock-associated MN activity (henceforth referred to as MN-photoinhibited mice) displayed normal escalating freezing responses to tones (Fig. 3*I*; two-way repeated-measures [RM] ANOVA, ArchT/control: *F*_(1,11)_ = 0.01, *P* = 0.9243). Behavioral responses during shocks (distance covered during shock-induced startle) were also not different between MN-photoinhibited and control mice (Fig. 3*J*; two-way RM ANOVA, ArchT/control: *F*_(1,11)_ = 1.18, *P* = 0.3005), suggesting that acute pain sensitivity was preserved. This was confirmed by photoinhibiting MNs during a hot plate test (32) (Fig. 3*K*; unpaired *t* test, *P* > 0.9999). In the context test, the freezing of the MN-photoinhibited mice was similar to that of control mice (Fig. 3*E*; two-way RM ANOVA, ArchT/control: *F*_(1,11)_ = 0.03, *P* = 0.8616), indicating that MN photoinhibition did not produce aberrant coupling of fear behavior to general context.

In contrast, across the fear extinction days, the MN-photoinhibited mice displayed significantly increased freezing compared with control mice (Fig. 3*L*; two-way RM ANOVA, ArchT/control: *F*_(1,11)_ = 10.16, *P* < 0.01). As expected from normal fear learning, the initial freezing to tone within extinction day 1 was similar in control and MN-photoinhibited mice (Fig. 3*F*). However, while control animals rapidly decreased freezing during the now-safe (i.e., unaccompanied by shock) tone, indicating normal safety learning, the MN-photoinhibited animals maintained high freezing within extinction day 1, indicating resistance to extinction (Fig. 3*F*; two-way RM ANOVA, time: *F*_(5,55)_ = 9.94, *P* < 0.001; ArchT/control: *F*_(1,11)_ = 6.609, *P* < 0.05; interaction: *F*_(5,55)_ = 4.432, *P* < 0.01). The abnormally high freezing in MN-photoinhibited mice persisted during extinction day 2, normalizing during extinction day 3 (Fig. 3*G*; two-way RM ANOVA, ArchT/control: *F*_(1,__11)_ = 7.778, *P* < 0.05; Fig. 3*H*: two-way RM ANOVA, ArchT/control: *F*_(1,__11)_ = 4.689, *P* = 0.0532). Collectively, these data suggest that endogenous MN activity during fear conditioning is not required for acquiring fear but is required for facilitating subsequent safety learning.

### Role of MNs in Relapsing Chronic Fear.

**Fig. 4.**
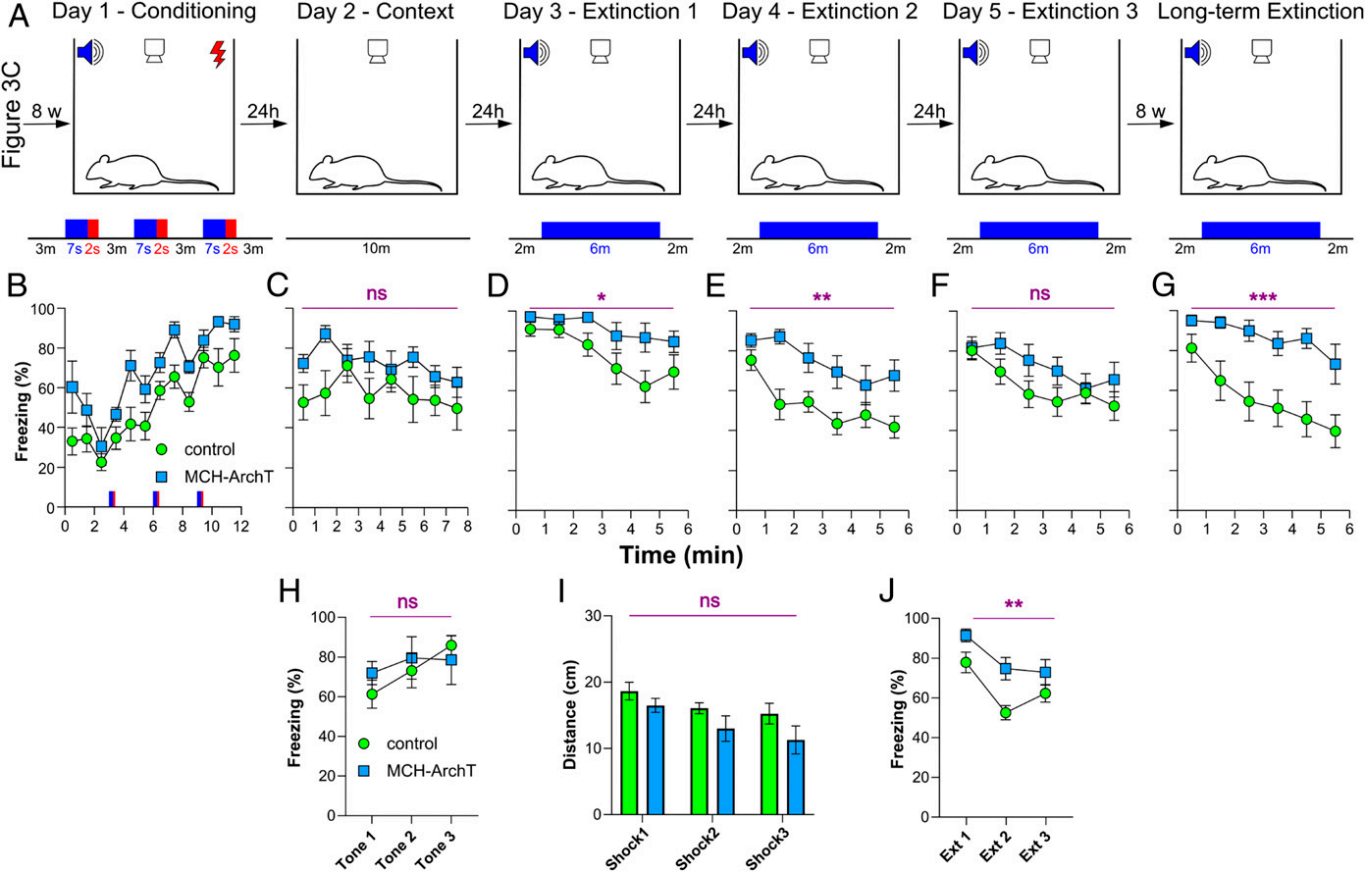
Role of MNs in chronic and relapsing fear behavior. (*A*) Protocol of fear reconditioning (the initial conditioning with MN photoinhibition is shown in Fig. 3*C*). (*B*–*F*) Chronic effects of MN photoinhibition on freezing corresponding to test phases shown in *A*. Each point shows a 60-s average (30 s before and 30 s after the point). (*G*) MN photoinhibition produces chronic, relapse-prone, dysfunctional fear extinction. (*H*) Chronic effect of MN photoinhibition on freezing during each 7-s tone on the conditioning day. (*I*) Chronic effect of MN photoinhibition on distance moved during conditioning shocks. ns, *P* > 0.1. (*J*) Chronic effect of MN photoinhibition on mean freezing during each 6-min extinction session on extinction days 1–3. Data are mean ± SEM of seven MN-ArchT mice and six control mice. ns, *P* > 0.05; *P < 0.05; **P < 0.01; ***P < 0.005 for ArchT vs. control. More details on the statistical tests are provided in *Results*.

We next sought to examine whether MNs could be involved in chronic, recurring symptoms typical of PTSD. To achieve this, we exposed the MN-photoinhibited and subsequently fear-extinguished mice (Fig. 3) to a second round of fear conditioning without further MN photoinhibition (Fig. 4*A*). This procedure, known as fear reconditioning, has been developed to induce reinstatement of the fear response to study fear relapse, a treatment-resistant and mechanistically elusive feature of many anxiety disorders (33, 34).

During the fear reconditioning (Fig. 4*B*), behavioral responses to tones and shocks were similar in MN-photoinhibited and control mice (tone-induced freezing: two-way RM ANOVA, ArchT/control: *F*_(1,11)_ = 0.85, *P* = 0.3774; shock-induced mobility: two-way RM ANOVA, ArchT/control: *F*_(1,11)_ =2.36, *P* = 0.1331), Fig. 4 *H* and *I*). Context-induced freezing was slightly, but not significantly, increased in MN-photoinhibited mice (Fig. 4*C*; two-way RM ANOVA, ArchT/control: *F*_(1,11)_ = 3.58, *P* = 0.0849). In contrast, subsequent safety learning was significantly disrupted in MN-photoinhibited mice (Fig. 4*J*; two-way RM ANOVA, ArchT/control: *F*_(1,11)_ = 6.20, *P* < 0.05). Within-day data are shown in Fig. 4 *D*–*F*. This demonstrates that the MN photoinhibition induces a chronic deficit in safety learning. Moreover, we found that relapse into excessive fear behavior in MN-photoinhibited mice was observed after another 8 wk, even without reconditioning (Fig. 4*G*; two-way RM ANOVA, ArchT/control: *F*_(1,11)_ = 13.12, *P* < 0.005). The freezing levels of MN-photoinhibited mice (Fig. 4*G*) were similar to the first extinction shown in Fig. 4*D*, while the control animals extinguished at a much higher rate in Fig. 4*G*. Overall, these results indicate that endogenous MN activity during acquisition of fear is required to prevent relapsing into pathological fear behavior in the future.

## Discussion

This study demonstrates endogenous MN activation during fear learning and shows that this activation is necessary for subsequent prevention of overactive cued fear behavior. These findings are unexpected and cannot be inferred from existing studies of MNs. While it has been proposed that MNs can modulate a number of learned and innate behaviors as well as synaptic plasticity via their brain-wide projections and/or volume transmission of MCH, no previously reported evidence suggested their involvement in extinction of cued fear responses (20–25, 31, 35–39).

Individuals suffering from pathological fear frequently experience false alarms that cause them to perceive safe situations as dangerous. This overactive fear is perhaps the major defining component of PTSD, where exaggerated and inflexible coupling of no-longer danger-predictive cues to fear produces disabling behaviors in inappropriate situations (40–42). Exposure-based fear extinction is an important source of therapeutic benefit, but it often fails, for poorly understood reasons (43–46). Our results showing resistance to extinction can provide a good animal model for studying the symptoms of such persistent, dysfunctional fear and investigating the neural mechanisms underlying PTSD. Importantly, the mouse model specifically recapitulates pathological safety learning (i.e., defective plasticity of coupling of the now-safe cue to freezing) while preserving the useful fear learning (coupling between shock and freezing).

The learning curves during fear conditioning were normal in the MN-photoinhibited animals. Their initial fear responses at the start of “exposure therapy” (Figs. 3*F* and 4*D*) were also intact, confirming the recent finding that cued fear responses are preserved in MN-ablated mice (24). MN photoinhibition did not disrupt freezing to context after conditioning, and there were no differences in initial freezing rates in the chamber before conditioning.

The chronic resistance of MN-photoinhibited mice to fear extinction probably was not due to differences in MN-induced pain modulation, as behavioral reactivity to shock during conditioning was not altered (Figs. 3*J* and 4*I*). In addition, the extinction-resistant fear observed in these animals cannot be attributed to a general fear sensitization due to increased pain or to a chronic generalized stress or arousal state (pain sensitivity tested in Fig. 3*K*; spontaneous locomotor activity, body weight, and anxiety levels tested in *SI Appendix*, Fig. S3 *A*–*D*). This has similarities to PTSD, which is resistant to exposure therapy, the clinical analog of extinction (47).

Our findings indicate new directions for further study of the neural circuits that underlie fear. At the systems level, the necessity of shock-associated MN signals for subsequent fear extinction suggests that they supply some nonredundant control signals. Thus, it would be of interest to determine what nonredundant information MN signals communicate among the multiple brain signals activated by traumatic events. At the cellular level, several mechanisms have been proposed to explain how a brief period of neural activity can chronically modulate the potential for future learning—for example, the synaptic tagging hypothesis (48). It has been proposed that in the period when a new fear association is formed, the consolidation process is labile during the first minutes following the aversive conditioning (49, 50). Therefore, disruption of MNs during conditioning might induce overconsolidation (51), leading to the strengthening of the aversive memory and in turn to impaired extinction. Usually fear learning is stronger than extinction learning (11, 52), and the threat memory can return even when extinction is initially successful, which may partially explain the relapse observed after several extinction sessions. The relationship of the shock-associated MN signals to such mechanisms is currently unknown and is an important subject for future work. At a translational level, our work provides an animal model for further understanding of neural machinery of fear disorders and advancing their treatments.

In conclusion, this study demonstrates that traumatic event-associated MN activity is mandatory for normal extinction of cued fear behavior. Given that MNs are present in the human brain (21), our findings may offer new insight into the pathophysiology of persistent anxiety and impaired extinction in disorders such as PTSD.

## Methods

### Genetic Targeting.

All animal procedures were performed in accordance with the Animal Welfare Ordinance (TSchV 455.1) of the Swiss Federal Food Safety and Veterinary Office and were approved by the Zurich Cantonal Veterinary Office. Mice were kept on a standard chow and water ad libitum and on a reversed 12-h/12-h light/dark cycle. Experiments were performed during the dark phase. Adult males (at least 8 wk old) were used for experiments.

The specific targeting of GCaMP6s and ArchT to MCH neurons was performed using the same genetic tools as described and histologically validated in our previous study (25). In brief, to target optogenetic inhibitory actuator ArchT to MCH neurons, we injected Cre-dependent AAV1.Flex-ArchT-GFP (4.6 × 10^12^ gc/mL; UNC Vector Core) into the lateral hypothalamus of the previously characterized and validated MCH-Cre mice (53), which were bred in het-WT pairs with C57BL/6 mice. Confirmation of functional ArchT expression (Fig. 3*B*) was performed using whole-cell patch clamping combined with photostimulation in acute brain slices (25). To target GCaMP6s to MCH neurons, we injected an AAV vector carrying the 0.9-kb preproMCH gene promoter AAV9.pMCH.GCaMP6s.hGH (1.78 × 10^14^ gc/mL; Vigene Bioscience, characterized to target MCH cells with >90% specificity in ref. 25) into the lateral hypothalamus of C57BL6 mice. Consequently, >90% MCH cells (280 of 302 cells from three brains) expressed GCaMP6s when analyzed using the histology protocol described previously (25). Patch-clamp recordings from transgene-expressing and WT MCH neurons (*SI Appendix*, Fig. S3*E*), including identification of WT MCH neurons by postrecording immunolabeling, was performed as described previously (54). For stereotaxic brain injections, mice were anesthetized with isoflurane and injected with carprofen (5 mg/kg of body weight, s.c.) for analgesia. In a stereotaxic frame (Kopf Instruments), a craniotomy was performed, and a 33-gauge needle mounted on a Hamilton syringe was used to inject AAV vectors into the hypothalamus. Three injections (each 50 nL, at a rate of 50 nL/min) were administered per hemisphere at the following coordinates: bregma, −1.35 mm; midline, ±0.90 mm; depth, 5.70 mm, 5.40 mm, and 5.10 mm (25, 28). Before the experiments, the mice were allowed to recover from surgery for at least 10 d.

### Fiber Photometry and Optogenetics.

Fiber optic implants were stereotaxically installed with the fiber tip above the lateral hypothalamus (bregma, −1.35 mm; midline, ±0.90 mm; depth, 5.00 mm) and fixed to the skull (25,28, 55). Fiber photometry was performed using the Doric fiber photometry system, in lock-in mode using simultaneous illumination with two LEDs (405-nm and 465-nm excitation, oscillating at 334 and 471 Hz, respectively; average power, ∼100 μW at the fiber tip). Fluorescence produced by 405-nm excitation provided a real-time control for motion artifacts (29). To produce the plotted % ΔF/F values, the raw 405-nm–excited signal was fitted to the 465-nm–excited signal, then the % ΔF/F time series was calculated for each session as [100*(465 signal – fitted 405 signal)/fitted 405 signal], based on ref. 56. No animals were excluded from analysis, but two plots (Fig. 2 *B* and *C*, right plots) contained six out of seven mice due to a recording equipment malfunction.

In the photoinhibition experiments, a green laser (532 nm; Laserglow Technologies) was connected to the bilateral fiber implants to yield ∼10 mW light power output at the fiber tip. Since photometry recordings showed that endogenous MCH signals were activated shortly after shock onset and persisted for no longer than 25 s, the laser illumination was applied for 25 s at shock onset.

### Fear Conditioning and Extinction.

The test was carried out in an operant chamber (model E10-10; Coulbourn Instruments) installed in a ventilated, sound-insulated chest and equipped with a grid floor made of stainless steel rods (4-mm diameter). Scrambled electric shocks with a 0.5 mA intensity were delivered through the grid floor (model E13-14; Coulbourn Instruments). Tones (2.9 kHz, 90 dB) were delivered through intrachamber speakers. The chamber had a total floor area of 30 cm × 25 cm and a height of 29 cm, but the mouse was confined to a rectangular 17.5 cm × 13 cm region in the center, defined by a clear Plexiglas enclosure. Sessions were recorded with a built-in IR camera (30 fps), and an automated video tracing system (Noldus EthoVision XT) was used to analyze freezing behavior.

### Hot Plate Test.

The animal was placed in a transparent glass cylinder atop an electrical hot plate (MEDAX; 13801). The temperature of the hot plate was set at 52 °C (±1°), and paw withdrawal latency (Fig. 3*K*) was defined as the time between when the animal was placed on the hot plate surface and when it licked its hind paw or jumped to avoid thermal pain (32).

### Elevated Plus Maze.

The elevated plus maze contained two open and two enclosed arms (each 6 cm × 35 cm), connected through a central square (6 cm × 6 cm), elevated 75 cm above the floor, in a dimly lit room. Mice were placed in the central square and allowed to move freely for 5 min. Sessions were recorded and analyzed using an automated system (Noldus EthoVision XT). Assessment of anxiety behavior was calculated as follows: 100*(time spent in open arms)/(time spent in open arms + time spent in closed arms).

### Data Analysis.

Statistical tests and descriptive statistics were performed as specified in *Results* and the figure legends. Mice were excluded from analysis if freezing before tone presentation on extinction 1 was >75%; this resulted in the exclusion of one control mouse in MN-photoinhibition experiments. Data are presented as mean ± SEM, and a *P* value < 0.05 was considered to indicate significance. Peak responses were calculated by subtracting the highest value of a 7-s pretone baseline period from the highest value of a 7-s period starting at stimulus (tone or shock) onset or offset. To compare behavior in ArchT and control mice, RM ANOVA was used, with multiple comparison tests where appropriate. Analysis was performed using GraphPad Prism 8 and MATLAB R2019b (MathWorks).

## Supplementary Material

Supplementary File

## Data Availability

All study data are included in the main text and *SI Appendix*.
