# Supplementary material for: Control of fear extinction by hypothalamic melanin-concentrating hormone–expressing neurons
Source: Proc Natl Acad Sci U S A. 2020 Aug 26;117(36):22514–21. doi: 10.1073/pnas.2007993117 (PMC7486764; doi:10.1073/pnas.2007993117)
Supplement: Supplementary File [file pnas.2007993117.sapp.pdf]

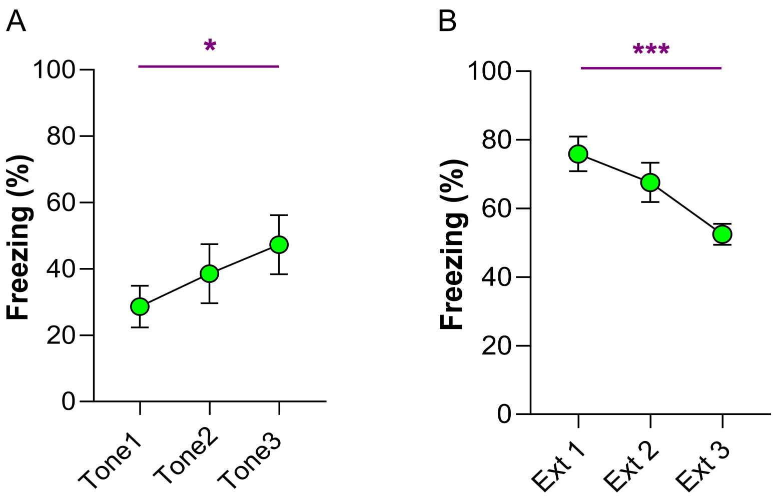

Figure S1. *Additional analysis of behavioral data from Figure 1.*
A. Mean freezing during each 7 sec tone on the conditioning day (one-way RM ANOVA, time: $F(2,12) = 4.205$, $P = 0.0413$). **B.** Mean freezing during each 6 min extinction session (one-way RM ANOVA, time: $F(2,10) = 25.14$, $P = 0.0001$). Data are means ± s.e.m of n = 7 MN-GCaMP6s mice.

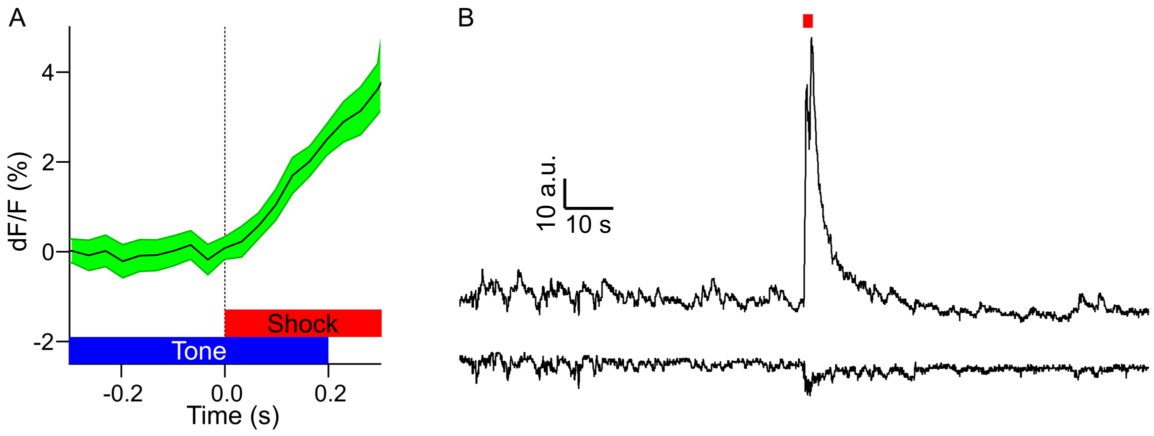

Figure S2. *Additional aspects of data in Figure 2.*
A. Shock-onset-associated rise in MN-GCaMP6s activity, at high temporal resolution (means ± s.e.m of n = 7 mice). **B.** Example photodetector response of a single shock-induced MN-GCaMP6s fluorescence response, top 465 nm excitation, bottom control 405 nm excitation; red, shock.

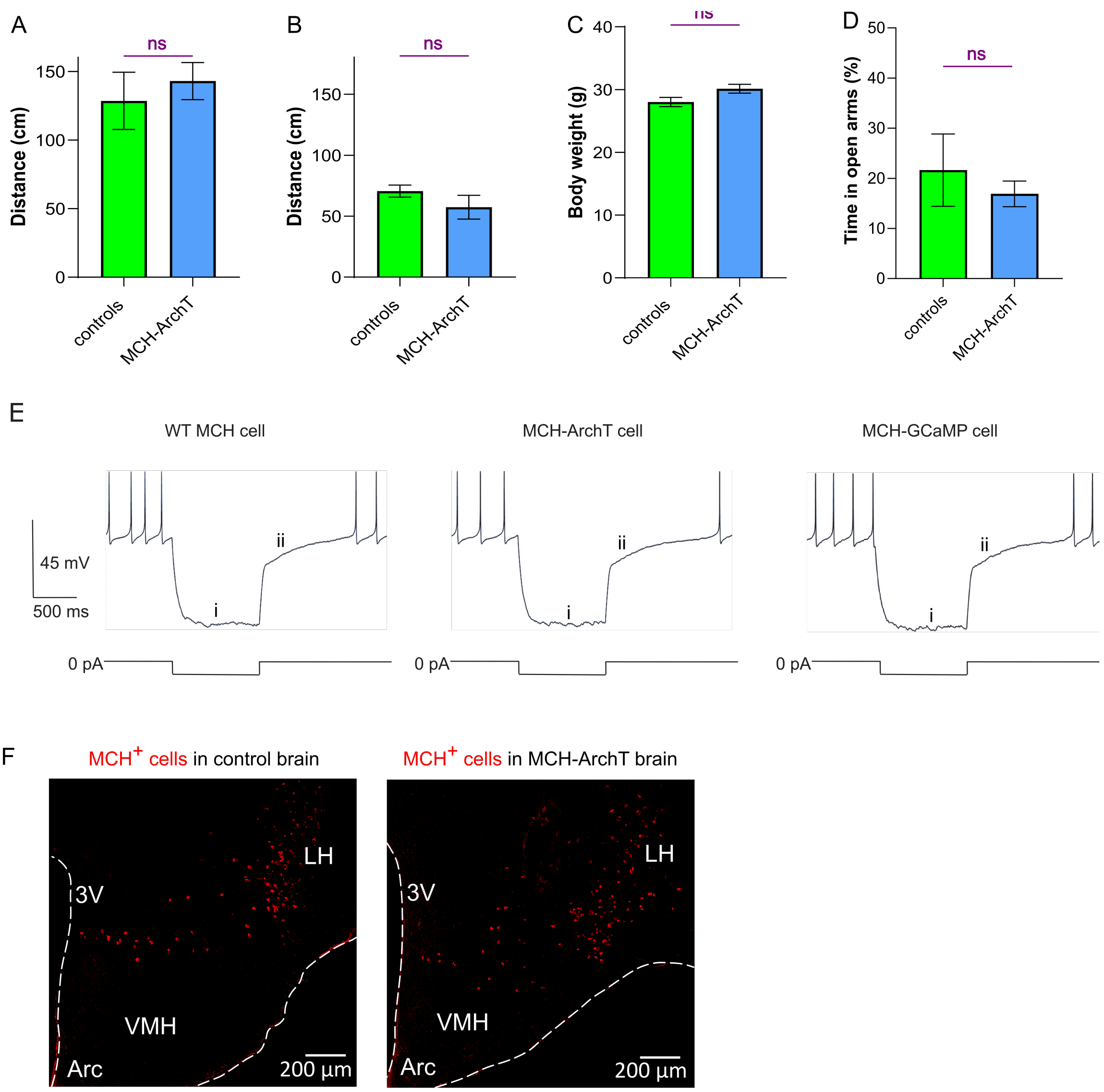

Figure S3. *Control experiments relating to data in Figures 3 and 4.*
A. Distance moved during 5 min before fear conditioning in Figure 3D (unpaired t test, $P = 0.2304$). **B.** Distance moved during 5 min before fear reconditioning and in Figure 4B (unpaired t test, $P = 0.2637$). **C.** Body weights of MCH-ArchT and control mice at the end of experiments in Figure 4B-F (unpaired t test, $P = 0.0571$). Data are means ± s.e.m of n = 7 MN-ArchT and n = 6 control mice. **D.** % time spent in open arms of the elevated plus maze (unpaired t test, $P = 0.6240$), performed after the end of experiments in Figure 4B-F. Data are means ± s.e.m of n = 4 MN-ArchT and n = 6 control mice. **E.** Patch-clamp recordings from acute brain slices, illustrating similar electrical fingerprints, such as absence of membrane sag upon hyperpolarizing current injection (i) and postinhibitory hyperpolarisation (ii) in WT (n = 19 cells), MCH-ArchT (n = 8 cells) and MCH-GCaMP (n = 9 cells) neurons. Transgene-expressing MCH cells were recorded 12 weeks after AAV injection and WT MCH cells from mice of a similar age. Spontaneous firing rates of WT MCH-GCaMP and MCH-ArchT cells were similar to WT MCH cells (MCH-GCaMP and WT MCH cells: means ± s.e.m. = 3.9 ± 1 Hz and 3.8 ± 1 respectively, unpaired t test $P = 0.9964$; firing of MCH-ArchT and WT MCH cells: 3.75 ± 1 Hz and 3.8 ± 1 respectively, unpaired t test $P = 0.9146$). **F.** Confirmation of MCH expression in brain slices from control and MCH-ArchT mice from Figure 4. Cell counts in n = 11 MCH-ArchT and 11 control sections (50 µm sections -1.35 to -1.80 from Bregma, from n = 2 ArchT and n = 3 control brains): mean ± s.e.m. = 76.45 ± 11.76 vs 83.18 ± 4.89 cells respectively, unpaired t test, $P = 0.6031$. Slices were labelled with a rabbit antibody to pMCH (M8440, 1:500, Sigma-Aldrich) and a Cy3-conjugated donkey antibody to goat IgG (705-165-147, 1:500, Jackson ImmunoResearch).
